# A uniform survey of allele-specific binding and expression over 1000-Genomes-Project individuals

**DOI:** 10.1038/ncomms11101

**Published:** 2016-04-18

**Authors:** Jieming Chen, Joel Rozowsky, Timur R. Galeev, Arif Harmanci, Robert Kitchen, Jason Bedford, Alexej Abyzov, Yong Kong, Lynne Regan, Mark Gerstein

**Affiliations:** 1 Program in Computational Biology and Bioinformatics, Yale University, New Haven, Connecticut 06520, USA; 2 Integrated Graduate Program in Physical and Engineering Biology, Yale University, New Haven, Connecticut 06520, USA; 3 Department of Molecular Biophysics and Biochemistry, Yale University, New Haven, Connecticut 06520, USA; 4 Department of Computer Science, Yale University, New Haven, Connecticut, 06520, USA; 5 Keck Biotechnology Resource Laboratory, Yale University, New Haven, Connecticut 06511, USA

## Abstract

Large-scale sequencing in the 1000 Genomes Project has revealed multitudes of single nucleotide variants (SNVs). Here, we provide insights into the functional effect of these variants using allele-specific behaviour. This can be assessed for an individual by mapping ChIP-seq and RNA-seq reads to a personal genome, and then measuring ‘allelic imbalances' between the numbers of reads mapped to the paternal and maternal chromosomes. We annotate variants associated with allele-specific binding and expression in 382 individuals by uniformly processing 1,263 functional genomics data sets, developing approaches to reduce the heterogeneity between data sets due to overdispersion and mapping bias. Since many allelic variants are rare, aggregation across multiple individuals is necessary to identify broadly applicable ‘allelic elements'. We also found SNVs for which we can anticipate allelic imbalance from the disruption of a binding motif. Our results serve as an allele-specific annotation for the 1000 Genomes variant catalogue and are distributed as an online resource (alleledb.gersteinlab.org).

## Abstract (web summary)

Using variants from the 1000 Genomes Project, RNA-seq and ChIP-seq data from related projects, this study describes a resource and survey of allele-specific binding and gene expression. A catalogue of allelic SNPs and annotation elements is available as an online resource at alleledb.gersteinlab.org.

## Graphical abstract

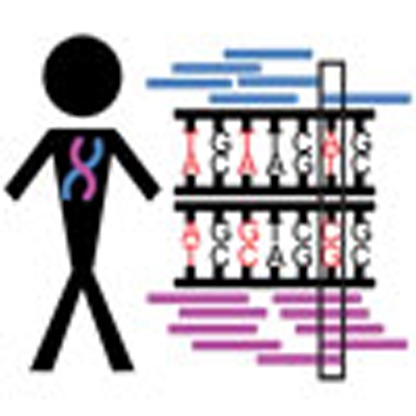

In recent years, the number of personal genomes has increased markedly, from single individuals^1^,^2^ to large sequencing projects such as the 1000 Genomes Project^3^ and UK10K (ref. 4). These efforts have provided the scientific community with a massive catalogue of human genetic variants, most of which are rare^3^. Subsequently, a major challenge is to functionally annotate these variants.

Much of the characterization of variants so far has been focused on those found in the protein-coding regions, but the advent of large-scale functional genomic assays, such as chromatin immunoprecipitation sequencing (ChIP-seq) and RNA sequencing (RNA-seq), has facilitated the annotation of genome-wide variation. This can be accomplished by correlating functional readouts from the assays to genomic variants, particularly in identifying regulatory variants, such as mapping of expression quantitative trait loci^5^,^6^,^7^ and allele-specific^8^,^9^ variants. Expression quantitative trait loci mapping assesses the effects of variants on expression profiles across a large population of individuals and is usually used for detection of common regulatory variants. On the other hand, allele-specific approaches assess phenotypic differences directly at heterozygous loci within a single genome. Using each allele in a diploid genome as a perfectly matched control for the other allele, allele-specific variants can be detected even at low population allele frequencies. Therefore, allele-specific approaches are very powerful, in terms of functionally annotating personal genomes, especially for identifying rare cis-regulatory variants on a large scale.

Early high-throughput implementations of allele-specific approaches used microarray technologies, and thus are restricted to a small subset of loci^10^,^11^. Later studies have used ChIP-seq and RNA-seq experiments for genome-wide measurements of allele-specific variants but have been mostly limited to a single assay with a variety of individuals^12^, or a few individuals with deeply sequenced and well-annotated genomes^9^,^13^. For instance, GM12878, a very well-characterized lymphoblastoid cell-line from a Caucasian female, has several RNA-seq data sets and a huge trove of ChIP-seq data for >50 transcription factors (TFs) distributed across multiple studies^14^,^15^. Merging these data sets to create a database is advantageous. The database consolidates a catalogue of annotated allele-specific variants in a central repository. Data sets belonging to the same individuals can also be combined to increase statistical power in detection and to have more features for intra- and inter-individual comparisons (such as having more TFs and populations or investigating allele-specific binding (ASB) and expression (ASE) coordination).

However, it is not optimal to simply aggregate results from multiple studies, even for the same biological sample. This is because disparate studies might design RNA-seq and ChIP-seq experiments with various goals in mind. Even if allele-specific analyses are conducted, they are often performed with different methods and sets of tools, parameters and variations of the same test (Supplementary Table 1). In addition, each allele-specific analysis is also sensitive to the technical issues associated with variant calling and processing RNA-seq and ChIP-seq experiments, such as thresholding and read mapping^16^,^17^. For example, homozygous single nucleotide variants (SNVs) incorrectly called as heterozygous will result in reads mapping to one allele (over the other), giving rise to false signals of allelic imbalance. Variants called using shorter reads such as those in RNA-seq data sets can also contain many artefacts. Thus, it is important to have a call set, particularly obtained from whole-genome DNA sequencing, such as those from the 1000 Genomes Project. Also, allele-specific SNVs detected in copy number variants have a higher rate of false positives, since copy number changes can easily masquerade as allelic imbalance.

Therefore, the task of merging has to be carried out in a uniform, standardized fashion to yield interpretable results. To this end, we organize and unify data sets from eight different studies into a comprehensive data corpus and repurpose it especially for allele-specific analyses. Our approach takes into account several issues. We alleviate allelic mapping bias via the use of personal genomes and the filtering of reads that give rise to ambiguous mapping. We also take the overdispersion of each individual data set into consideration when we harmonize them, and then a second time on the pooled data sets when we detect allele-specific variants. We also filter variants that are found in copy number variants. Overall, we detect more than 6K and 63K SNVs associated with ASB and ASE events, respectively, over 382 individuals from the 1000 Genomes Project. We are able to present a survey for these allele-specific variants in various general and specific categories of coding and non-coding genomic elements and annotations (for example, coding regions and enhancers) in a population-aware manner. We identified genomic regions that are enriched or depleted in allelic activity. Finally, using our consolidated data, we investigate the extent of purifying selection in allele-specific SNVs and the inheritance of allele-specific expression and ASB in two different TFs. The variants and annotations are available as an online resource, AlleleDB (http://alleledb.gersteinlab.org/).

## Results

### AlleleDB Workflow

**Figure 1:**
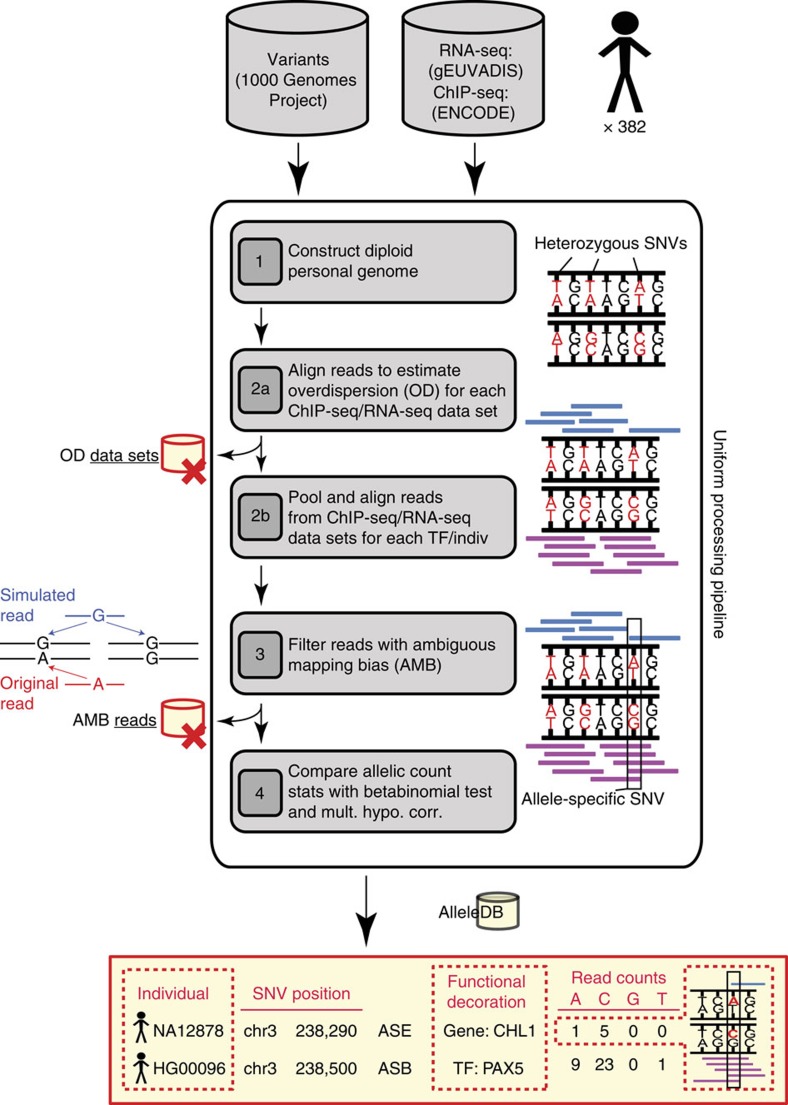
Workflow for uniform processing of data from 382 individuals and construction of AlleleDB. For each of the 382 individuals, (1) a diploid personal genome is first constructed using the variants from the 1000 Genomes Project. Next, reads from individual (2a) and pooled (2b) ChIP-seq or RNA-seq data sets are mapped onto each of the haplotypes of the diploid genome. In (2a), overdispersion (OD) is measured for each data set and used to flag and segregate highly overdispersed data sets (‘OD data sets'). (2b) The resultant data sets are pooled. (3) From each pooled read pile for each individual and each TF (for ChIP-seq data sets), original reads that give rise to simulated ambiguous-mapping reads are removed (‘AMB reads'). (4) For each filtered read pool, we estimate an OD parameter and then detect allele-specific SNVs. For detection, we compare the read counts between the two parental chromosomes and a statistical significance is computed (after multiple hypothesis test correction) based on the beta-binomial test using the ‘pooled' OD parameter to account for OD. All the candidate allele-specific variants are then deposited in AlleleDB database. Additional information, such as raw read counts of both accessible non-allele-specific and allele-specific variants, can be downloaded for further analyses.

**Figure 2:**
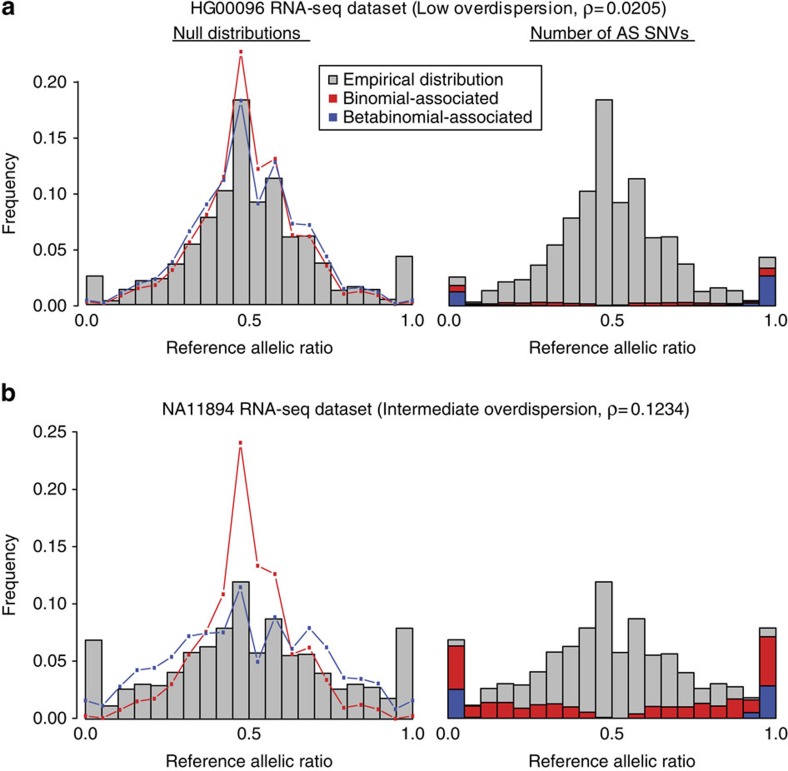
Comparing the effects of the binomial and beta-binomial tests in data sets with low and intermediate level of overdispersion. The grey bars in each plot represent the empirical allelic ratio distribution. For **a** and **b**, the red and blue lines on the left plots represent the null (expected) allelic ratio distributions associated with the binomial and beta-binomial tests, respectively. The red and blue bars on the right plots represent the number of allele-specific (AS) SNVs detected in each for the binomial and beta-binomial tests, respectively. (**a**) The plots for one of the RNA-seq data sets for the individual HG00096. It has a low overdispersion parameter, ρ=0.0205. The empirical distribution does not have heavy tails and the binomial and beta-binomial tests give very similar results. (**b**) The plots for one of the RNA-seq data sets for the individual NA11894. Overdispersion is higher at ρ=0.1234, and the beta-binomial null distribution provides a better fit to the empirical allelic ratio distribution than the binomial distribution. The empirical distribution (grey bars) also show heavier tails, signifying more SNVs with allelic imbalance.

In general, the AlleleDB workflow uniformly processes two pieces of information from each individual: the DNA variants, and reads from either the ChIP-seq or RNA-seq experiment to assess SNVs associated with ASB or ASE, respectively (Fig. 1). (1) It starts by first constructing a diploid personal genome for each of the 382 individuals, using DNA variants from the 1000 Genomes Project. (2) It then aligns the ChIP-seq or RNA-seq data set to each of the haploid genomes instead of the human reference genome, and chooses the better uniquely mapped alignment. This reduces reference bias that can potentially result in erroneous read mapping^13^. Because each individual can have multiple ChIP-seq or RNA-seq data sets, the alignment is performed to each personal genome twice. (2a) In the first round, the alignment is performed for each of 276 ChIP-seq and 987 RNA-seq data sets to calculate a measure of overdispersion (with respect to an expected binomial distribution) ‘ρ' (see the ‘Methods' section). We observed that if there is a greater overdispersion in the empirical allelic ratio (defined as the proportion of reads that map to the reference allele) distribution of a data set, the binomial test tends to overestimate the number of allele-specific events (Fig. 2). There are varying degrees of overdispersion in our data sets, even between biological replicates. In general, RNA-seq data sets are generally more consistent in overdispersion than ChIP-seq data sets. Differing overdispersion in individual data sets poses a challenge later in Step 2b when we pool and merge multiple data sets. To harmonize the data sets, we flag and filter data sets that are deemed to be more overdispersed in allelic ratio distributions, leaving us with 186 ChIP-seq and 955 RNA-seq data sets for allele-specific detection (Supplementary Table 2). (2b) The second alignment is performed by ‘pooling' the 186 ChIP-seq and 955 RNA-seq data sets that have not been filtered in Step 2a. The pooling is performed for each individual and each TF (for ChIP-seq); for example, CTCF (CCCTC-binding factor) ChIP-seq data sets for NA12878 that were not filtered were pooled together. An overdispersion parameter is re-calculated for each pooled set. (3) The third module filters reads that exhibit a bias that we term as ‘ambiguous mapping bias' (AMB). This bias occurs at a locus when reads containing one allele are preferred, not because of better alignments, but because of sequence homology in the region overlapping the other allele, with another location in the genome. As a result, reads with the other allele align ambiguously to multiple locations and are consequently removed, resulting in an erroneous allelic imbalance at that locus (Fig. 1). This module detects reads that exhibit AMB via simulations. Briefly, for each original uniquely mapped read (we call the ‘O read') that overlaps at least one heterozygous SNV on one parental genome, we simulate reads (‘S reads') that represent all possible haplotypes of that O read. We then align the S reads to the other parental genome. ‘O' reads with ‘S' reads that map to multiple locations (we call them ‘AMB reads') are filtered from the aligned reads obtained in Step 2b (see Fig. 1 and the ‘Methods' section). (4) Finally, we obtain allelic counts from the filtered read pile and estimate a ‘pooled' overdispersion parameter. Beta-binomial tests are performed using the ‘pooled' overdispersion parameter to detect allele-specific SNVs. For ChIP-seq data, the SNVs are further pared down to those within peak regions. We also remove SNVs if they lie in regions predicted to be copy number variants. Please refer to the ‘Methods' section for a more detailed description.

**Figure 3:**
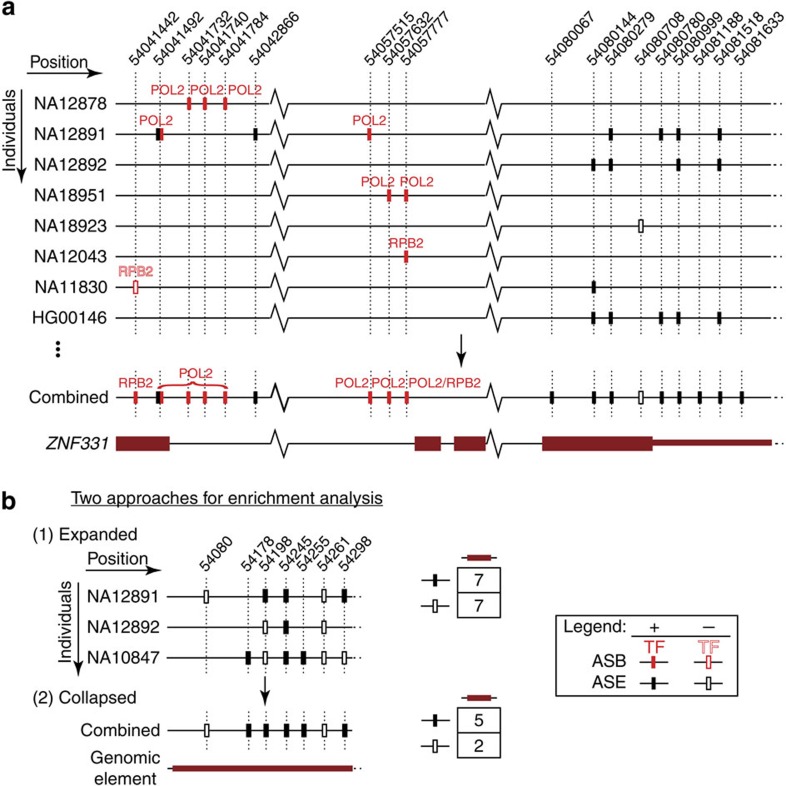
Part of the *ZNF331* gene on chromosome 19, position 54,041,442-54,081,633 (hg19). (**a**) ASB and ASE SNVs in allele-specific gene *ZNF331*. From AlleleDB, we can observe the ASB SNVs (filled red bars with the name of the TF above the bars) and ASE SNVs (filled black bars) found in each individual (row) and genomic positions (columns) along the *ZNF331* gene. We can see that many of these SNVs are sparsely distributed across a single individual. By collapsing or combining information from multiple individuals, we can identify genomic regions or elements that are enriched for allele-specific activity. Unfilled black and red bars denote control SNVs are heterozygous SNVs that have enough reads to be tested but are non-allele-specific. (**b**) Two approaches for enrichment analyses are performed for each genomic element. (1) The ‘expanded' enrichment is performed in a population-aware fashion, in which each occurrence of allele-specific or control non-allele-specific SNV in each individual is counted. (2) The ‘collapsed' enrichment conflates all occurrences over multiple individuals into a single unique SNV position as long as an allele-specific or accessible non-allele-specific SNV occurs in at least one individual.

We built a database, AlleleDB (http://alleledb.gersteinlab.org/), to house the annotations, the allele-specific and accessible SNVs. AlleleDB can be downloaded as flat files or queried and visualized directly as a UCSC track in the UCSC Genome browser^18^ as specific genes or genomic locations. This enables cross-referencing of allele-specific variants with other track-based data sets and analyses, and makes it amenable to all functionalities of the UCSC Genome browser. Heterozygous SNVs found in the stipulated query genomic region are colour-coded in the displayed track; Fig. 3a shows a schematic that illustrates an example of a visualization. Such visualization allows ASB and ASE to be viewed together conveniently. By building the resource using the individuals and variants from the 1000 Genomes Project, AlleleDB also serves as an allele-specific annotation of the 1000 Genomes Project variant catalogue (Supplementary Data 1 and 2).

### ASB and ASE inheritance analyses using CEU trio

**Figure 4:**
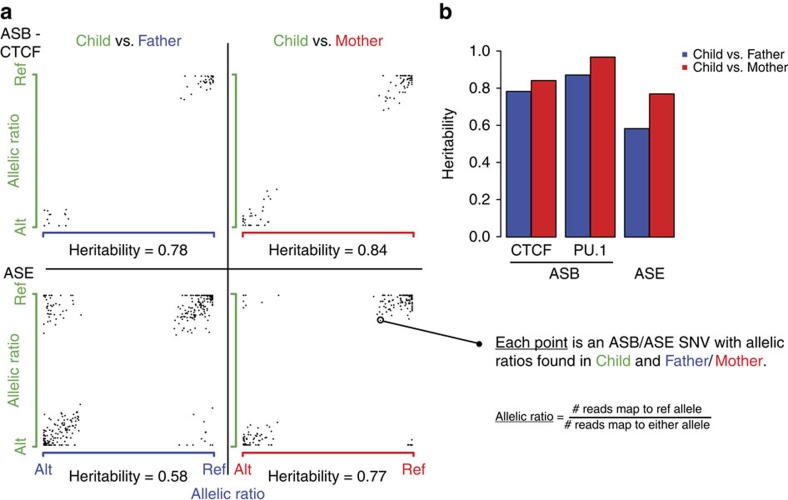
Inheritance of allele-specific behaviour. (**a**) The left panel shows plots for the TF CTCF (top row) and ASE (bottom row) being examined for inheritance in the CEU trio (Father: NA12891, blue; Mother: NA12892, red; Child: NA12878, green). Each point on the plot represents the allelic ratio of a common ASB SNV between the parent (*x*-axis) and the child (*y*-axis), by computing the proportion of reads mapping to the reference allele at that SNV. High Pearson's correlations, *r*, observed in both parent-child comparisons for CTCF (*r*≥0.78) signify strong heritability in allele-specific behaviour. ASE also shows considerably strong evidence of heritability but has comparatively lower *r* values. (**b**) The bar plot at the top right panel presents the r values for ASB in two TFs and ASE in our analyses.

The CEU trio is a well-studied family and with multiple ChIP-seq studies performed on different TFs. Previous studies have also presented allele-specific inheritance^9^,^14^,^19^. Here, after uniformly processing data sets from multiple studies, we are able to analyse and compare the heritability of ASE and ASB across two DNA-binding proteins in a consistent manner (Fig. 4; see the ‘Methods' section). For the DNA-binding protein CTCF and PU.1 (also SPI1, or spleen focus forming virus proviral integration proto-oncogene), we observed a high parent–child correlation (Fig. 4, Supplementary Table 4), denoting great similarity in allelic directionality (Pearson's correlation, *r*≥0.78 in both parent–child plots). We also observed considerable heritability in ASE, but to a lesser degree. In general, the high inheritance of allele-specific SNVs observed in the same allelic direction from parent to child also implies a sequence dependency in allele-specific behaviour.

### AS variants and enrichment analyses

Of great interest is the annotation of allele-specific SNVs with respect to known genomic elements, both coding and non-coding. We calculate the enrichment of ASB and ASE SNVs in various genomic categories. To do so, we have to define ‘accessible' and ‘control' SNVs. Accessible SNVs are heterozygous SNVs that have at least the minimum number of reads needed to be statistically detectable for allelic imbalance, which is calculated independently for each data set (see the ‘Methods' section).

We further define the control SNVs as the non-allele-specific subset of accessible SNVs, by excluding ASB or ASE SNVs from each set of accessible SNVs. Thus, the control SNVs are non-allele-specific but are matched in terms of the minimum number of reads to the detected allele-specific SNVs (see the ‘Methods' section). This matching is especially pertinent to our enrichment analyses, since the Fisher's exact test is dependent on the choice of the null expectation (that is, controls).

Using the AlleleDB variants found in the personal genomes of the 2 parents of the trio and 379 unrelated individuals from Phase 1 of the 1000 Genomes Project, we focus on autosomal SNVs and detected 63,541 unique ASE and 6,136 ASB SNVs, representing 14% and 6% of the accessible SNVs, respectively (Tables 1 and 2). In all, 16% of our candidate ASE SNVs and 6% of ASB SNVs are in the coding DNA sequences (CDSs); these correspond to log odds ratios of −0.2 (depletion) and 0.3 (enrichment), when compared with the non-coding regions (Supplementary Fig. 1).

To estimate the degree of allele-specificity in both coding and non-coding genomic elements, we calculate the enrichment of allele-specific SNVs by comparing allele-specific SNVs relative to the control SNVs using Fisher's exact tests. The enrichment analyses are performed in two ways: ‘expanded' and ‘collapsed'. The former counts each occurrence of SNV in a population-aware manner, where each control or allele-specific SNV is counted for each individual at each locus. The latter collapses and counts a control or allele-specific SNV location as a unique SNV, as long as it occurs in at least one individual (Fig. 3b). Both enrichment analyses are performed in genomic annotations (or categories) with differing granularities, from broad genomic categories to individual binding motifs and genes. Broad genomic categories are grouped on the basis of similar functional context. These include 708 non-coding genomic categories from the ENCODE project^20^ (for example, DNaseI hypersensitivity sites and TF-binding motifs) and six gene sets known to be involved in monoallelic expression^21^,^22^ (for example, imprinted genes^23^, olfactory receptor genes^24^).

We further calculate the enrichment of allele-specific SNVs in 19,257 autosomal protein-coding genes from GENCODE^25^ in both collapsed and population-aware expanded fashion. The database allows us to visualize allele-specific SNVs across the gene region and over multiple individuals. For example, *SNRPN* and *SNURF* are maternally imprinted genes, shown to be highly implicated in the Prader–Willi Syndrome, an imprinting disorder^26^. Indeed, they are two of our most highly ranked allele-specific genes by overall odds ratio (column ‘AS.OR' in Supplementary Data 3). When *SNURF* is queried in our database, we can see clearly that the allele-specificity is supported not only by evidence from 55 ASE loci across the gene but also by the fact that a number of variants are shown to be also allele-specific over multiple individuals, one variant even up to 160 individuals. The concurrent visualization of ASB and ASE SNVs with respect to genomic elements using the UCSC genome browser is also another advantage of AlleleDB (Fig. 3a). For example, *ZNF331* (zinc-finger protein 331) gene contains both ASE and ASB SNVs. It has previously been shown experimentally to be consistently expressed from the paternal allele^27^. Our visualization shows ASB loci from POL2 (RNA polymerase II largest subunit) and RPB2 (RNA polymerase II second largest subunit) in several individuals coinciding near *ZNF331* exons; these two DNA-binding proteins are components of RNA polymerase II (Fig. 3a).

**Figure 5:**
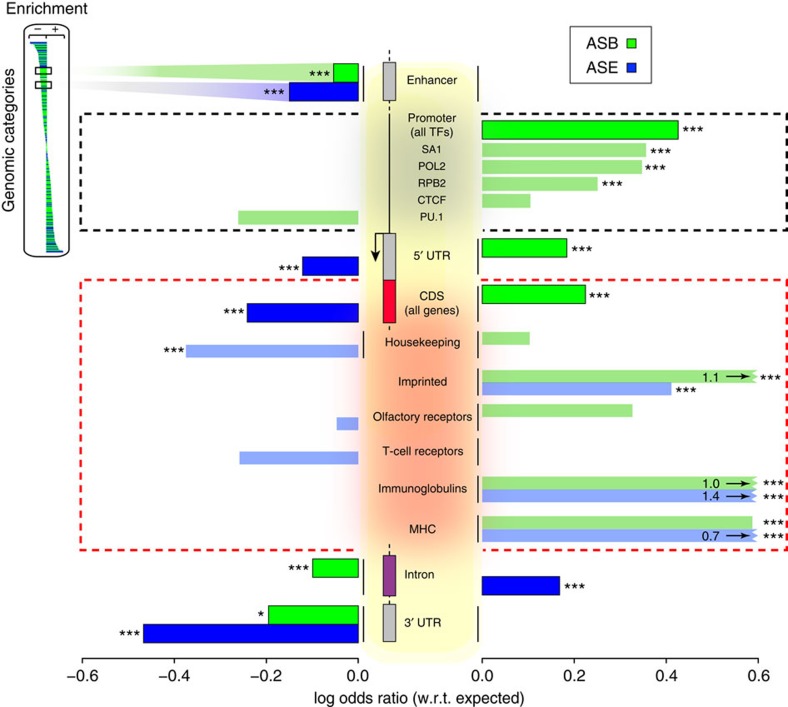
Population-aware 'expanded' enrichment analysis shows that some genomic regions are more inclined to allele-specific regulation. The ‘expanded' analysis is performed in a population-aware manner, where each control or allele-specific SNV is counted once for each occurrence in an individual. We map variants associated with ASB (green) and ASE (blue) to various categories of genomic annotations, such as CDSs, UTRs, enhancer and promoter regions, to survey the human genome for regions more enriched in allelic behaviour. Using the control non-allele-specific SNVs as the expectation, we compute the log odds ratio for ASB and ASE SNVs separately, via Fisher's exact tests. Bonferroni-corrected: *P<0.05; **P<0.01; ***P<0.001. For each TF in AlleleDB, we also calculate the log odds ratio of ASB SNVs in promoters, providing a proxy of allele-specific regulatory role for each available TF. Genes known to be mono-allelically expressed such as imprinted and MHC genes (CDS regions) are highly enriched for both ASB and ASE SNVs. The actual log odds ratio of ASB SNVs in imprinted genes, both ASB and ASE SNVs in immunoglobulin genes and ASE SNVs for MHC is indicated on the bar.

In addition, we extend the enrichment analyses to gene elements, such as introns and promoter regions. Figure 5 and Supplementary Fig. 1 shows the enrichment of allele-specific SNVs in elements closely related to a gene model, namely enhancers, promoters, CDSs, introns and untranslated regions (UTRs). For SNVs associated with ASB, we observed an enrichment in the 5′-UTRs. This is in-line with an enrichment of ASB SNVs in promoters, suggesting functional roles of these variants in regulating gene expression. We see variable enrichments of ASB SNVs in the peaks of particular TFs such as POL2, SA1 (cohesin subunit) and CTCF in promoter regions, while depletion in others, such as PU.1 (Fig. 5, Supplementary Data 4). These differences might imply that some TFs are more likely to participate in allele-specific regulation than others. Between the two enrichment analyses, we observe more consistent trends in the odds ratios of ASB SNVs than ASE SNVs. The differences are most likely contributed by the presence of common SNVs that are also behaving consistently (either being allele-specific or non-allele-specific) over multiple individuals.

The population-aware analysis gives additional power to calculate enrichment for very specific genomic annotations, namely specific protein-coding genes, enhancers and TF-binding motifs; this is unlike broad genomic categories that span over multiple regions in broad genomic categories. By computing the enrichment analysis in a population-aware fashion, we can also define elements based on evidence supported over multiple individuals. This allows us to quantify allele-specific consistency and enrichment even within smaller and specific protein-coding genes (Supplementary Data 3), and enhancers (Supplementary Data 5), and differentiate those annotations that are significantly and more consistently enriched to be ‘allele-specific', depleted to be ‘balanced' or otherwise ‘indeterminate'. We provide these lists on the AlleleDB resource (http://alleledb.gersteinlab.org/download/).

### Rare variants and purifying selection in AS SNVs

To assess the occurrence of ASB and ASB SNVs in the human population, we consider the population minor allele frequencies (MAFs). Tables 1 and 2 show the breakdown of the accessible and allele-specific SNVs in six ethnic populations (we combined the results for CHB and JPT) and allele frequencies. Yoruba from Ibadan, Nigeria (YRI) contribute the most to both ASE and ASB variants at each allele frequency category. The number of rare allele-specific SNVs (MAF≤5%) is about twofold higher in the YRI than the other European sub-populations of comparable (CEU, FIN) or larger (TSI) population sizes (see the ‘Methods' section for full explanation of population abbreviations). However, the percentage of allele-specific SNVs (in accessible SNVs) remains fairly consistent. In general, rare variants do not form the majority of all the allele-specific variants. For each category of allele frequency, the proportion of allele-specific SNVs detected (with respect to accessible SNVs) is fairly comparable across populations (CEU, FIN, GBR, TSI and YRI), with a slight enrichment of ASB SNVs and slight depletion of ASE SNVs as we go towards lower frequencies, while the proportion remains fairly consistent in a single individual (Tables 1, 2, 3).

**Figure 6:**
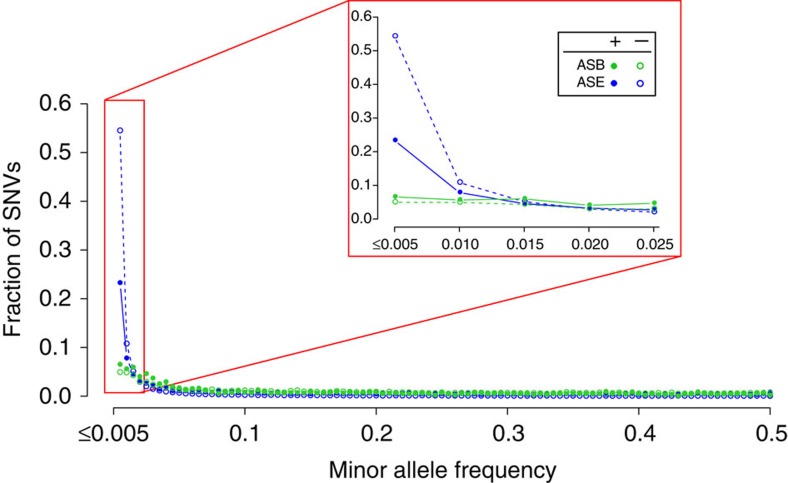
A considerable fraction of allele-specific variants are rare but do not form the majority. A lower proportion of allele-specific SNVs than non-allele-specific SNVs are rare, suggesting less selective constraints in allele-specific SNVs. The MAF spectra of ASB (green filled circle), control non-ASB SNVs (green open circle), ASE (blue filled circle) and control non-ASE SNVs (blue open circle) are plotted at a bin size of 100. The peaks are in the bin for MAF≤0.5% (corresponding to 0.005 in Figure 6). The inset zooms in on the histogram at MAF≤2.5%. The proportion of rare variants in descending order: ASE−>ASE+>ASB+>ASB−. Comparing ASE+ with ASE− gives an odds ratio of 0.3 (Bonferroni-corrected hypergeometric *P*<2.2e−16), while comparing ASB+ with ASB−, gives an odds ratio of 1.3 (*P*=0.2), signifying statistically significant depletion of ASE SNVs but statistically insignificant enrichment of ASB SNVs relative to the respective non-allele-specific control SNVs. Statistically significant depletion in ASE suggests that ASE SNVs are under less purifying selection.

To examine selective constraints in allele-specific SNVs, we then consider the enrichment of rare variants with MAF≤0.5% (refs 3, 28). Figure 6 shows a shift of the allele frequency spectrum towards very low allele frequencies in all allele-specific and non-allele-specific SNVs, peaking at MAF≤0.5%. We limit our analyses for ASE SNVs to only those found in CDS regions and ASB SNVs to only those found within known TF motifs (among the 708 non-coding categories in Supplementary Data 6). In general, ASE SNVs are shown to have a greater enrichment of rare variants than ASB SNVs. This is probably due to the background of ASE SNVs being in genes versus ASB SNVs mostly in non-coding regions of the genome. Our results in Fig. 6 show a statistically significant lower enrichment of rare variants in ASE SNVs as compared with non-ASE SNVs (Fisher's exact test odds ratio=0.3, *P*<2.2e−16), but statistically insignificant higher enrichment of rare variants in ASB SNVs than non-ASB SNVs (Fisher's exact test odds ratio=1.3, *P*=0.2). This observation suggests that ASE variants may be under weaker selection than non-ASE variants.

### AS variants in TF-binding motifs affecting TF occupancy

A pertinent ASB analysis is to identify ASB SNVs that might cause a TF-binding difference. To perform this analysis, we focus on the 277 ASB SNVs found across multiple individuals that reside in the binding motifs of 15 TFs. We consider an allele to be disruptive when it occurs less frequently at a position in the motif. Thus, we compare the difference in occurrence between the reference and the alternate allele of the ASB SNV in the position weight matrix (PWM) of a TF-binding motif. For instance, if the alternate allele is disruptive, the reference allele is favoured, and the difference in occurrence>0 (see the ‘Methods' section). We then correlate this with the allelic ratio at the ASB SNV. We expect a TF-binding motif that favours the reference allele of an ASB SNV (difference in occurrence>0) to be associated with more binding to the reference allele (that is, allelic ratio>0.5). We find a statistically significant correlation between the difference in occurrence and the allelic ratio for the 277 ASB SNVs (Pearson's correlation=0.78, *P*<2.2e−16), showing that there is indeed an overall trend for the favoured allele to correspond to increased TF binding. In general, the effects of the SNVs are consistent across individuals in the context of the same motifs. As a resource, we provide the list of ASB SNVs with the frequencies of the occurrence of their reference and alternate alleles found in the various TF motifs and their corresponding allelic ratios (Supplementary Data 7).

## Discussion

The binomial test is typically used to provide statistical significance for the identification of allele-specific SNVs. However, previous studies have observed a deviation from the binomial distribution in read-count distributions in ChIP-seq and RNA-seq data sets, which in turn results in broader allelic ratio distributions, that is, overdispersed^5^,^29^,^30^. The beta-binomial test introduces additional parameters to account for overdispersion. Data sets with low overdispersion give very similar results between binomial and beta-binomial tests (Fig. 2a). The binomial test tends to overestimate the number of detected allele-specific SNVs in data sets with higher overdispersion, giving rise to more false positives (Fig. 2b). In addition to accounting for the overdispersion in the statistical inference of allele-specific SNVs, we propose the use of the overdispersion parameter, ρ, as a means of quality control for flagging data sets that are very different in the spread of the null allelic ratio distributions. This is because high overdispersion can in fact also serve as a strong indicator for potential issues in the data sets, such as uneven and/or sparse read coverage. Hence, while overdispersion could be a biological consequence of allele-specific behaviour, we typically assume that allelic ratios of most loci are balanced. The removal of ‘outlier' data sets then facilitates the process of homogenizing and harmonizing the data sets. Consequently, we propose the utility of overdispersion as both a means of data set quality control and allele-specific SNV detection in a beta-binomial test.

Another source of error that we investigated and accounted for is the allelic mapping bias. This occurs when one allele is preferentially aligned over the other allele in read alignment, resulting in the detection of erroneously imbalanced SNVs. In this study, we have accounted for two types of mapping biases, namely reference bias and AMB.

A personal-genome-based approach has been cited as one of the more rigorous but computationally intensive approaches in reference bias reduction^13^,^16^,^31^,^32^,^33^. In addition, the personal genome is able to handle various mapping artefacts not easily managed by using only the reference genome. In particular, the personal genome is able to incorporate larger variants beyond SNVs (such as indels), making it a more representative genome of the individual, which was previously demonstrated by Rozowsky *et al.*^13^ and Sudmant *et al.*^34^, where the personal genome is shown to give a much better alignment of unique reads.

The second allelic mapping bias stems from loci with sequence homology, or AMB. There have been a number of strategies developed to deal with the AMB (Supplementary Table 1). Our AlleleDB implementation of a read-removal strategy has the dual advantage of removing false positives and yet retaining robust allele-specific SNVs, as compared with the more stringent site-removal strategy. Interestingly, this removal of reads has also been used very recently by van de Geijn *et al.*^35^. We note that the ambiguous mapping is also highly dependent on the length of the read, as shown by Degner *et al.*^17^, with the bias decreasing with increasing read length. We envision that AMB will be further alleviated by long read technologies being used in functional assays.

It is important to note that the AS SNVs detected are still not necessarily causal. The resultant allelic difference in gene expression and binding can still be due to another undetected causal variant that has a strong linkage disequilibrium with the detected variant or, it could be due to a group of variants that act collectively to give the resultant allelic expression or binding^36^. It could also be a result of other epigenetic effects such as genomic imprinting where no genetic variants are causal^37^. Nonetheless, the computational detection of allele-specific SNVs is still useful by allowing us to prioritize variants on a large scale in terms of their potential impact. For example, we provide a more confident set of allele-specific SNVs, since they are found to be in the same allelic direction supported by evidence in at least three individuals in AlleleDB (Supplementary Data 8). We also provide a list of high-impact ASB SNVs that cause a change in the PWMs of the TF-binding motifs (Supplementary Data 7).

So far, allele-specific analyses have usually been more SNV- or gene-centric. However, many diseases have been found to also implicate allelic activity in non-genic genomic regions^38^,^39^. The quantification of allele-specific activity in known genomic elements and annotations, such as CDSs and various non-coding regions, will not be feasible without a large number of ASE and ASB SNVs. Moreover, a significant portion of AS SNVs is rare, thus the abundance and detection of rare AS variants will increase with many genomes. Previous studies mostly focus on a very small number of genomes, making it difficult to perform AS analyses from a single study. A possible solution, as we have shown here, is to pool and process data sets and genomes from multiple studies uniformly. Such data pooling enables the aggregation of information from multiple SNVs (both rare and common) across a genomic element, thereby allowing the large-scale characterization of allele-specific activity in genomic elements. Consolidating rare allele-specific SNVs is also helpful in defining SNV sets, which allows us to assign allelic activity scores to genomic regions or multiple variants based on allele-specific activity; this is akin to the idea of burden tests for rare variants in association studies^40^,^41^. Such an assignment of allelic activity scores is useful when incorporating into large-scale annotation pipelines^42^.

We have adopted two ways to analyse enrichment: an expanded approach that capitalizes on the number of individuals and a collapsed approach that computes enrichment based on unique allele-specific SNVs occurring in at least one individual. An expanded population-aware approach highlights the corroborating effects of common allele-specific variants in an element found across multiple genomes. On the other hand, a collapsed approach treats each common and rare variant independently. An element that is deemed more allelic in this case but not in the population-aware enrichment analysis, might mean that there are more rare variants exhibiting allele-specific behaviour. This type of allele-specific elements would not have been picked out with a small number of genomes. Thus, a difference in results from the two analyses of the same element can suggest an interplay between rare and common allele-specific SNVs.

In addition, we can provide some insights into the coordination of ASB and ASE within that category, by comparing ASB and ASE enrichments within specific genomic regions or broad categories (Fig. 5). For example, loci that are associated with monoallelic expression have been shown to be also associated with ASB of various TFs, such as imprinted^43^,^44^ and immunoglobulins genes^45^. Also, in Fig. 3a, we can visualize, in AlleleDB, specific sub-regions within the *ZNF331* gene where ASB and ASE coordination might occur.

In conclusion, there is great value in integrating existing data, especially across a large collection of genomes that are accurate and diverse. Better personal genome construction will improve read alignment and subsequent allele-specific SNV detection. Several studies have already used long read technologies to enhance personal genomes with more accurate haplotype information^46^,^47^. Genome diversity is also important in the annotation of rare variants. Our current catalogue of allele-specific SNVs is detected from lymphoblastoid cell lines, which is the predominant cell-line type in the literature. To capture the variability in regulation of gene expression in different tissues^48^, data from projects, such as GTEx^49^, can contribute valuable functional assays and sequencing of other tissues and cell lines. Furthermore, our search for data sets shows a dearth of ChIP-seq and RNA-seq data sets with corresponding personal genomes in non-European populations. Since many allele-specific variants have been found to be rare at both the individual and the sub-population level, it is of great interest and importance that more individuals of diverse ancestries be represented—a concern echoed previously in population genetics^50^. In the future, large-scale genome annotation will definitely benefit from increasingly accurate data sets of diverse provenance.

## Methods

### Construction of diploid personal genomes

There is a total of 382 genomes used in this study: 379 unrelated low-coverage genomes (average depth of 2.2 to 24.8) from Utah residents in the United States with Northern and Western European ancestry (CEU), Han Chinese from Beijing, China (CHB), Finnish from Finland (FIN), British in England and Scotland (GBR), Japanese from Tokyo, Japan (JPT), Toscani from Italy (TSI) and Yorubans from Ibadan, Nigeria (YRI) and three high-coverage genomes from the CEU trio family (average read depth of 30 × from Broad Institute's GATK Best Practices v3; variants are called by UnifiedGenotyper). Each diploid personal genome is constructed from the SNVs and short indels (both autosomal and sex chromosomes) of the corresponding individual found in the 1000 Genomes Project. This is constructed using the tool, *vcf2diploid*^13^. Essentially, each variant (SNV or indel) found in the individual's genome is incorporated into the human reference genome, hg19. Most of the heterozygous variants are phased in the 1000 Genomes Project; those that are not, are randomly phased. As a result, two haplotypes for each individual are constructed. When this is applied to the family of the CEU trio, for each child's genome, these haplotypes become the maternal and paternal haplotypes, since the parental genotypes are known. Subsequently, at a heterozygous locus in the child's genome, if at least one of the parents has a homozygous genotype, the parental allele can be known. However, for each of the genomes of the 379 unrelated individuals and the 2 parents from the CEU trio, the alleles, though phased, are of unknown parental origin.

CNV genotyping is also performed for each personal genome by CNVnator^51^, which calculates the average read depth within a defined window size, normalized to the genomic average for the region of the same length. For each low-coverage genome, a window size of 1,000 bp is used, while for the high-coverage genomes, a window size of 100 bp is used. SNVs found within genomic regions with a normalized abnormal read depth <0.5 or >1.5 are filtered out, since these would mostly likely give rise to spurious allele-specific detection.

We use the personal genomes to alleviate reference bias, which occurs when the read with the reference allele is more favourably mapped. This is especially observed in the alignment to the human reference genome because the read with the alternate allele has already at least one mismatch to begin with. Since reads are typically aligned to the haploid human reference genome in conventional allele-specific analyses, the reference bias has been widely regarded as the main source of allelic mapping bias^16^,^17^,^33^. There have been a number of strategies developed to alleviate reference bias^31^; we provide some examples in Supplementary Table 1.

The personal genomes and additional resources can be accessed from the AlleleDB website (http://alleledb.gersteinlab.org/).

### RNA-seq and ChIP-seq data sets

In total, we reprocessed 287 ChIP-seq for 14 individuals and 993 RNA-seq data sets for 382 individuals from 8 different studies (Supplementary Table 2).

RNA-seq data sets are obtained from the following: gEUVADIS^12^, ENCODE^20^, Lalonde *et al.*^52^, Montgomery *et al.*^53^, Pickrell *et al.*^5^, Kilpinen *et al.*^14^ and Kasowski *et al.*^15^.

ChIP-seq data sets are obtained from the following: ENCODE^20^, Kilpinen *et al.*^14^, Kasowski *et al.*^15^ and McVicker *et al.*^54^.

### Read alignment and estimation of overdispersion ‘ρ'

Reads are aligned against each of the derived haploid genome (maternal/paternal genome for trio) using Bowtie 1 (ref. 55). When a read is aligned to the same locus, we only pick the alignment that maps better to a haplotype. Otherwise, if a read is tied in alignment to both haplotypes, we keep that read and randomly assign the read to a haplotype. No multi-mapping is allowed and only a maximum of two mismatches per alignment is permitted. This enables the calculation of the proportion of reads that align to the reference allele, or the allelic ratio, at each heterozygous SNV.

To estimate ρ, we adopt a three-step approach. We first obtain the empirical histogram for the allelic ratios of all heterozygous SNVs with read counts≥6. Next, we calculate the expected null distribution (where there is no allelic imbalance) using the probability density function of the beta-binomial distribution using the R package, VGAM^56^:


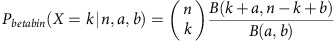


where, *n* represents the total number of reads at a particular locus, *B*(x,y) represents the beta function with variables x and y, *a* and *b* represent the shape parameters of the beta distribution. For computational efficiency, if *n*≥1,000, we set it to a maximum of 1,000, but retain the allelic ratio at the SNV. The VGAM beta-binomial routines require the input of the overdispersion parameter, ρ, and probability of success (also the mean of the beta distribution), which we fix at 0.5 since the null hypothesis assumes no allelic imbalance. We then obtain the expected beta-binomial distributions for ρ=0 to ρ=1 with an increment of 0.1, and choose ρ that minimizes the least sum of squared errors (LSSE) between the empirical and the expected distributions. Finally, to further refine our estimate, we iterate a bisection method to arrive at a LSSE (R pseudo-code available in Supplementary Data 9).

After removing 11 ChIP-seq and 6 RNA-seq data sets that had insufficient read alignments, we calculated ρ for each 276 ChIP-seq and 987 RNA-seq individual data sets. For RNA-seq data sets, we removed 32 data sets with ρ≥0.125, which is 1 standard deviation higher than the mean ρ amongst the RNA-seq data sets. For ChIP-seq data sets, because many of the data sets have considerable ρ, we use a less stringent arbitrary threshold of ρ≥0.3 to remove 90 ChIP-seq data sets. Using the resultant 186 ChIP-seq and 955 RNA-seq data sets, we pooled data sets by TF and individual for ChIP-seq and by individual for RNA-seq. Each pooled set is then processed to remove reads with AMB (described in next section). ρ is then re-calculated for each filtered pooled data set. This final ρ is used in the beta-binomial test for allele-specific SNV detection.

### Accounting for AMB

AMB (ambiguous mapping bias) is termed so, because reads from one allele might align ambiguously to multiple locations, resulting in reads with the other allele being unduly favoured (Fig. 1)^17^,^31^,^35^. Several strategies have been implemented in dealing with the AMB (Supplementary Table 1). To date, the primary approach has been the identification and removal of sites in which >5% of the total number of reads exhibit such bias^12^,^31^,^33^,^49^. In our study, we observed that many detected SNVs remain allele-specific even after removing reads that display such bias, showing that the site removal strategy can be overly conservative (Supplementary Table 5). Hence, we remove reads, instead of sites, that exhibit AMB.

More specifically, (1) we first align the reads to each of the two parental haplotypes of the diploid personal genome of each individual (and each TF for ChIP-seq). (2) For each haplotype, we retain only those reads that uniquely mapped to regions with heterozygous SNVs. For a uniquely mapped read that overlap at least one heterozygous SNV on one parental genome (‘O' read), we simulate reads that represent all possible haplotypes of that ‘O' read (‘S' reads). For example, for ‘O' reads that overlap a single heterozygous SNV, ‘S' reads are the same reads but with a single allele change at the heterozygous SNV position (Fig. 1). If the ‘O' read overlaps multiple heterozygous SNVs, ‘S' reads of all other possible haplotypes are simulated. Due to computational complexity and higher probability of harbouring sequencing errors, we remove ‘O' reads that overlap >5 heterozygous SNVs; Supplementary Table 3 shows that the probability of getting such ‘O' reads is very small. (3) We then map all ‘S' reads to the other parental genome. (4) Finally, we identify and filter the ‘O' reads which give rise to ‘S' reads that align to multiple loci in the other parental genome or do not map back to the same location; we consider ‘O' reads to exhibit AMB. We also exclude any ‘O' reads in which neither of the alleles of the overlapping SNVs matches the nucleotide on the corresponding read, as they suggest sequencing errors. Apart from the AlleleDB implementation, we also introduced an alternative approach into the AlleleSeq pipeline to account for AMB, where we explicitly check for multi-mapping reads at loci with allelic imbalance (details are given in Supplementary Note 1).

### Allele-specific SNV detection

Allele-specific SNV detection is performed on the pooled data sets, as mentioned above. Here, a beta-binomial *P* value is derived based on the VGAM R package, as described in the previous section. Similarly for computational efficiency, if *n*≥1,000, we set it to a maximum of 1,000, but retain the allelic ratio at the SNV. To correct for multiple hypothesis testing, FDR is calculated. Since statistical inference of allele-specificity of a locus is dependent on the number of reads of the ChIP-seq or RNA-seq data set, this is performed using an explicit computational simulation^13^. Briefly, for each iteration of the simulation, a mapped read is randomly assigned to either allele at each heterozygous SNV and a beta-binomial test is performed using the estimated ρ. At a given *P* value threshold, the FDR can be computed as the ratio of the number of false positives (from the simulation) and the number of observed empirical positives. An FDR cutoff of 10% is used for ChIP-seq data and 5% for RNA-seq data, since the latter is typically of deeper coverage. Furthermore, significant allele-specific SNVs have a minimum of six reads.

For ChIP-seq data, allele-specific SNVs have to be also within peaks. Peak regions are determined by first performing PeakSeq^57^ for each of the 14 personal haploid genomes with ChIP-seq data. Only a single read per strand per position is kept and duplicates removed. The fragment length is set to 200 bp. Peak calling is performed with default parameters and the final peak set for each TF is identified at an FDR of 5%. Finally, the coordinates of the peaks (based on the respective personal haploid genomes) are mapped to the reference genome coordinates using the UCSC genome browser tool LiftOver^18^, and then finally being merged between the two haploid genomes. We also make the uniformly processed peaks available as a resource on the AlleleDB website.

Allele-specific detection has minimal bias towards sites with lower read depth (Supplementary Figs 2 and 3) and is highly reproducible when we compare between replicates (Supplementary Figs 4 and 5). The detection for all TFs and gene expression of 382 individuals took about 600 days in CPU time (1.6 years), but the pipeline is highly parallelizable, thereby streamlining the process.

### AlleleDB

The final data and results are organized into a resource, AlleleDB (http://alleledb.gersteinlab.org/), which conveniently interfaces with the UCSC genome browser for query and visualization; currently all variant coordinates are based on the human reference genome, hg19. Since many in the scientific community are familiar with the genome browser, we hope that this would increase the accessibility and usability of AlleleDB. The query results are also available for download in BED format, which is compatible with other tools, such as the Integrated Genome Viewer^58^. More in-depth analyses can be performed by downloading the full set of allele-specific results. For ASB, the output will be delineated by the sample ID and the associated TFs; for ASE, the output will be categorized by individual and the associated gene. We also provide the raw counts for each accessible SNV and indicate if it is identified as an allele-specific SNV. AlleleDB also serves as an annotation of allele-specific regulation of the 1000 Genomes Project SNV catalogue. All Supplementary Data and additional auxiliary materials can also be downloaded on AlleleDB via http://alleledb.gersteinlab.org/download/.

### Allele-specific inheritance analyses

The conventional measure of ‘heritability' allows the estimation of (additive) genetic contribution to a certain trait. The population genetics definition of ‘heritability' in a parent–offspring setting is described by the slope, β, of a regression (*Y*=β*X*+α), with the dependent variable being the child's trait value (*Y*) and the independent variable (*X*) being the average trait values of the father and the mother (‘midparent')^59^. This is a population-based measure typically performed on a large set of trios for a particular trait (for example, height) and β is not necessarily bound between 0 and 1.

Given that we have only a single trio, we adapt the typical definition of ‘heritability' to quantify allele-specific inheritance for each TF. For each TF and parent-child comparison, we consider ASB SNVs from two scenarios: (1) when an allele-specific SNV is heterozygous in all three individuals but common to the two individuals being compared, and (2) when an allele-specific SNV is heterozygous in two individuals and homozygous (reference or alternate) in the third. We define the allelic ratio as the ‘trait', which is a continuous value and computed as the proportion of reads that align to the reference allele with respect to the total number of reads mapped to either allele of a particular site. We perform the analyses separately for father–child and mother–child pair to maximize statistics, since a midparent calculation will require that a SNV is allele-specific in all three individuals (Scenario 1).

Given that Pearson's correlation coefficient, *r*, always gives a value between 0 and 1, we use *r* instead of β, as our measure of ‘heritability'. We also compute and include β-values in Supplementary Table 4. The parent–parent comparison is provided as a source of comparison for two unrelated individuals with shared ancestry. For parent–parent β, the maternal allelic ratio is chosen arbitrarily to be the independent variable.

### Genomic annotations

Categories of gene elements from Fig. 5 and Supplementary Fig. 1, such as promoters, CDS regions and UTRs, and 19,257 autosomal protein-coding gene annotations (HGNC symbols) are obtained from GENCODE version 17 (ref. 25). Promoter regions are set as 2.5 kbp upstream of all transcripts annotated by GENCODE.

Gene annotations also include 2.5 kbp upstream of the start of gene. In all, 708 categories of non-coding annotations are obtained from ENCODE Integrative release^20^, which includes broad categories such as TF-binding sites, and annotations such as distal-binding sites of particular TFs, for example, ZNF274. The details of TF family classification is first described in Vaquerizas *et al.*^60^ and then also in Gerstein *et al.*^61^ Note that these TF-binding sites are separate from those in promoter regions in Fig. 5 and Supplementary Fig. 1, which are based on the 40 TFs and peaks from the ChIP-seq experiments used in our pipeline.

The olfactory receptor gene list is from the HORDE database^24^; immunoglobulin, T-cell receptor and MHC gene lists are from IMGT database^62^. Imprinted genes are merged from the Catalog of Parent-of-origin Effects (http://igc.otago.ac.nz/home.html)^63^, the GeneImprint website (http://www.geneimprint.com/) and also Lo *et al.*^11^. We performed enrichment analyses on a number of enhancer lists, which are derived using the ChromHMM and Segway algorithms^64^,^65^, and data from distal regulatory modules from Yip *et al.*^66^. The result for the enhancers in Fig. 5 is based on the union of these lists (http://info.gersteinlab.org/Encode-enhancers). An additional enhancer list for experimentally validated enhancers is obtained from VISTA enhancer browser database^67^ (http://enhancer.lbl.gov/). Housekeeping gene list is obtained from Eisenberg and Levanon^68^ (http://www.tau.ac.il/~elieis/HKG/).

### Enrichment analyses

Enrichment analyses were performed in two ways: ‘collapsed' and ‘expanded' (Fig. 3b). In both cases, we aggregate ASB and ASE SNVs within a specific genomic element, such as a gene or an enhancer. We then use the Fisher's exact test to calculate the odds ratio and the hypergeometric *P* value, to test for the enrichment of allele-specific SNVs compared with ‘control' SNVs, which are non-allele-specific ‘accessible' SNVs. For these analyses, we only use the SNVs from unrelated individuals to prevent redundancy (that is, 379 unrelated individuals and either NA12878 or her parents; NA12878 SNVs are never used together with those from the parents, except in cases of some TFs where there are more NA12878 ASB SNVs, NA12878 ASB SNVs are used in lieu of the parents').

We define the set of accessible SNVs as all heterozygous SNVs that exceed the minimum number of reads required in order for SNVs to be significantly detectable by the beta-binomial test for each data set; this includes both allele-specific and non-allele-specific SNVs. This is an additional, more stringent criterion imposed beyond the minimum threshold of six reads. Given a fixed FDR cutoff, for a larger data set, the beta-binomial *P* value threshold is typically lower, making the minimum number of reads (*N*), which will produce the corresponding *P* value, larger. This alleviates a bias in the enrichment test for including SNVs that do not have sufficient reads in the first place. Considering an extreme allelic imbalance case where all the reads are found on one allele (all successes or all failures, that is, allelic ratio is 0 or 1), this minimum *N* can be obtained from a table of expected two-tailed beta-binomial probability density function, such that accessible SNVs are all SNVs with a minimum number of reads, *n*≥max(6,*N*). The minimum number of reads thus varies with the pooled size (coverage) of the ChIP-seq or RNA-seq data set. Thus, the accessible SNVs are data set-specific; they are determined for each pooled ChIP-seq (grouped by individual and TF, not by study) or RNA-seq data set (grouped by individual). By considering only the cases with the largest effect size, we underestimate the number of accessible SNVs and this provides a conservative approximation of the statistical significance of the enrichment (or depletion). ‘Control' SNVs are subsequently derived from accessible SNVs that are non-allele-specific, that is, they are the set of accessible SNVs that has excluded the respective ASB or ASE SNVs for each data set.

In the ‘collapsed' enrichment analysis, each control or allele-specific SNV is counted once uniquely, as long as it occurs in at least one individual in AlleleDB. The ‘expanded' analysis is performed in a population-aware manner, where each control or allele-specific SNV is counted once for each occurrence in an individual. *P* values are Bonferroni-corrected and considered significant if ≤0.05.

‘Allele-specific' and ‘balanced' autosomal protein-coding genes, enhancers and TF-binding motifs are defined based on statistically significant (Bonferroni-corrected *P* value≤0.05) enrichments (odds ratio≥1.5) or depletions (odds ratio<1.5), respectively, as obtained from the ‘expanded' enrichment analysis; the rest of the elements with non-significant odds ratios are considered ‘indeterminate'.

### SNV MAFs

The MAF spectra for Fig. 6 are obtained for accessible and allele-specific SNVs detected in samples in AlleleDB. We restrict our analyses for ASE SNVs for 381 unrelated samples (excluding NA12878) to only those found in CDS regions and ASB SNVs for 13 samples (excluding NA12878) to only those found within known TF motifs found in the 708 categories (Supplementary Data 6). The MAF of each SNV is a global MAF calculated with respect to 1,092 samples from Phase 1 of the 1000 Genomes Project^3^.

### Analysis of ASB SNVs found in TF motifs

We obtain a list of all TF motifs and their corresponding PWMs from Kheradpour and Kellis^69^ (http://compbio.mit.edu/encode-motifs/), using the 2013 version. This set of motifs and PWMs is derived from the ENCODE project and include motifs from TRANSFAC and JASPAR. We then take two approaches to find the effects of ASB SNVs. (1) For all ASB SNV positions in the motifs detected by Kheradpour and Kellis^69^, we obtain the occurrence (frequency) of their reference and alternate allele in the respective PWMs. This first approach is only able to find motif-breaking events that disrupt existing motifs in the reference genome. The PWMs of motifs are defined based on the ENCODE project. (2) Our second approach attempts to include both motif-breaking and motif-gaining events caused by ASB SNVs in AlleleDB. On the basis of each PWM, we further scan a 59-bp window around the ASB SNV (±29 bp of the SNV) separately for both the reference and alternate alleles for potential motifs. For each candidate motif, we compute the sequence score using the tool TFM-Pvalue^70^, where sequence score is defined by summing up the log likelihoods of each position of the PWM. A motif is identified when the *P* value on its sequence score≤1e−6.

We then merge the results from both approaches. The allelic ratio is defined as before, that is, the ratio of number of reference reads to the total number of reads, thus when the ratio>0.5, there are more reads that align to the reference allele, signifying more binding to the motif with the reference allele. We compute the difference in occurrence between the reference and alternate allele (occurrence of reference allele minus occurrence of alternate allele) based on the PWM of the motif, thus a positive value indicates that the reference allele is favoured (that is, less disruptive). The Pearson's correlation is calculated between this difference and the allelic ratio.

## Additional information

**How to cite this article:** Chen, J. *et al.* A uniform survey of allele-specific binding and expression over 1000-Genomes-Project individuals. *Nat. Commun.* 7:11101 doi: 10.1038/ncomms11101 (2016).

## Supplementary Material

Supplementary Information Supplementary Figures 1-5, Supplementary Tables 1-5 and Supplementary Note 1

Supplementary Data 1 This tab-delimited file contains all the ASB SNVs (autosomal) annotated by the 39 TFs and all individual samples used in our study. For each SNV, the raw counts, TF and sample annotation are provided. There is redundancy in the data due to SNVs occurring in multiple samples and TF binding sites. This file contains all SNVs (including those without MAF from 1000 Genomes Project) and all individuals (including the CEU trio).

Supplementary Data 2 This tab-delimited file contains all the ASE SNVs (autosomal) annotated by autosomal genes and samples used in our study. For each SNV, the raw counts, gene and sample annotation are provided. If a SNV is not in a gene, it is annotated as ‘NA' . There is redundancy in the data due to SNVs occurring in multiple samples and genes. This file contains all SNVs (including those without MAF from 1000 Genomes Project) and all individuals (including the CEU trio).

Supplementary Data 3 This Excel file contains results from our ‘collapsed' (_c) and ‘expanded' (_e) enrichment analyses for the 20,142 autosomal protein-coding genes (HGNC symbols) from GENCODE, including the Fisher' s exact test odds ratios (OR), original (p) and Bonferroni-corrected (p.bon) p-values, the number of allele-specific SNVs (AS, ASB, ASE) and accessible non-allele-specific (control) SNVs (nonAS, nonASB, nonASE) found in the gene region and the promoter region (upstream 2500bp). The results for housekeeping genes and 4 monoallelically-expressed (MAE) gene categories are also included. ‘NA' is marked in categories where odds ratio cannot be calculated due to insufficient numbers in non-allele-specific SNVs. These are tabulated for ASB, ASE and AS SNVs; the latter is the results for the combined number of ASB and ASE SNVs. There is an Excel tab with a legend explaining the abbreviations used. Based on the results with AS SNVs in the expanded (_e) analyses, we define gene regions that are “allele-specific” (Bonferroni p value ≤ 0.05, odds ratio ≥ 1.5), “balanced” (Bonferroni p value ≤ 0.05, odds ratio < 1.5) and otherwise “indeterminate”.

Supplementary Data 4 This Excel file contains the ASB ‘collapsed' (_c) and ‘expanded' (_e) enrichment analyses in promoter regions for 40 TFs used in our database, including Fisher' s exact test odds ratios (OR), original (p) and Bonferroni-corrected (p.bon) p-values, the number of allele-specific SNVs (only ASB) and accessible non-allele-specific (control) SNVs (nonASB) both found and not found (!Region) in the gene region. ASB SNVs for each TF are contributed by different individuals. If either of the parents in the CEU trio is involved, ASB SNVs for NA12878 are not included. Those TFs with only ASB SNVs from NA12878 are annotated ‘1' under the column ‘NA12878 only'. ‘NA' is marked in categories where odds ratio cannot be calculated due to insufficient numbers in any of the last three columns.

Supplementary Data 5 This Excel file contains the results from our ‘expanded' enrichment analysis for 882 experimentally-determined VISTA^67^ enhancers and 420,516 enhancer regions from the union of lists by Ernst and Kellis (2012)^64^, Hoffman et. al. (2013)^65^), and data from distal regulatory modules from Yip et al. (2012)^66^. The results include including the Fisher' s exact test odds ratios (OR), original (p) and Bonferroni-corrected (p.bon) p-values, the number of allele-specific SNVs (AS, ASB, ASE) and accessible non-allele-specific (control) SNVs (nonAS, nonASB, nonASE). ‘NA' is marked in categories where odds ratio cannot be calculated due to insufficient numbers in non-allele-specific SNVs. These are tabulated for ASB, ASE and AS SNVs; the latter is the combined number of ASB and ASE SNVs. Based on results in AS, we define enhancer regions that are “allele-specific” (Bonferroni p value ≤ 0.05, odds ratio ≥ 1.5), “balanced” (Bonferroni p value ≤ 0.05, odds ratio < 1.5) and otherwise “indeterminate”.

Supplementary Data 6 This Excel file contains results from our ‘collapsed' (_c) and ‘expanded' (_e) enrichment analyses for 708 categories from ENCODE, including the Fisher' s exact test odds ratios (OR), original (p) and Bonferroni-corrected (p.bon) p-values, the number of allele-specific SNVs (AS, ASB, ASE) and accessible non-allele-specific (control) SNVs (nonAS, nonASB, nonASE) found in each category. The results for five gene element categories from GENCODE and 16 enhancer categories are also included. ‘NA' is marked in categories where odds ratio cannot be calculated due to insufficient numbers in non-allele-specific SNVs. These are tabulated for ASB, ASE and AS SNVs; the latter is the results for the combined number of ASB and ASE SNVs. There is an Excel tab with a legend explaining the abbreviations used.

Supplementary Data 7 This Excel file contains the ASB SNVs that reside in TF motifs described in Kheradpour and Kellis69. The columns are respectively: chromosome (chr), 0-based start position (start) and 1-based end position of SNV (end), the reference (ref) and alternate (alt) allele, the frequencies of the reference (ref_freq) and alternate (alt_freq) allele in the position weighted matrix of the motif, the difference between these frequencies (diff_freq, with the reference frequency minus the alternate frequency), the motif identifier (motif), the transcription factor (TF), the individual sample identifier (indiv_TF) and the allelic ratio (allelicRatio). Under the column ‘motif' , the information is further delimited by “#” in this order: motif identifier (as defined in Kheradpour and Kellis), start position of motif (0-based), end position of motif (1-based), strand and position of SNV in motif. Allelic ratios at each SNV position are defined above, i.e. ratio of number of reference reads to number of alternate reads.

Supplementary Data 8 This Excel file contains sets of ‘more confident' ASB and ASE SNVs. The columns are respectively: chromosome (chr), start (0-based start), end (1-based end) position of the SNV, the TF and individual identifier (TF_ind in ASB or ind in ASE), number of individuals with this ASB SNV (indCount), and the allele with more reads (dominantAllele). For the 194 ASB SNVs, each of them is found in at least 3 individuals (indCount ≥ 3) and the allele that has more reads (dominantAllele) has to be the consistent for all TF_ind. For the more confident 1,890 ASE SNVs, each of them is found in at least 38 individuals (' indCount' ≥ 38). At the same time, for each of the SNV, the allele that has more reads (dominantAllele) has to be consistent in all the individuals (ind).

Supplementary Data 9 This Word file contains the R pseudocode for the bisection method that is used to estimate the overdispersion parameter.

## Figures and Tables

**Table 1:** Breakdown of ASE SNVs in each ethnic population.

**Population**	**Individual**	**SNVs**	**Total**	**Common SNVs (MAF>0.05)**	**Rare SNVs (MAF≤0.01)**	**Very rare SNVs (MAF≤0.005)**
CEU	75	HET	10,651,149	6,308,566	2,524,103	1,783,747
		ACC	272,764	211,466	31,357	21,794
		ASE	22,664 (8%)	16,347 (8%)	3,453 (11%)	2,546 (12%)
FIN	71	HET	10,004,674	6,241,404	2,048,207	1,401,508
		ACC	102,001	66,449	19,809	13,903
		ASE	14,276 (14%)	9,781 (15%)	2,718 (14%)	2,005 (14%)
GBR	67	HET	10,276,322	6,217,359	2,319,797	1,624,709
		ACC	110,901	71,257	22,735	16,326
		ASE	14,805 (13%)	10,220 (14%)	2,644 (12%)	1,996 (12%)
TSI	92	HET	11,475,093	6,331,145	3,104,327	2,293,577
		ACC	119,326	70,407	30,240	22,969
		ASE	18,364 (15%)	12,027 (17%)	3,691 (12%)	2,723 (12%)
YRI	74	HET	17,173,494	6,202,905	7,189,972	4,887,934
		ACC	217,253	93,225	74,924	49,923
		ASE	32,484 (15%)	16,191 (17%)	9,690 (13%)	6,685 (13%)
CHB/JPT	2	HET	3,185,252	2,892,601	114,990	76,669
		ACC	27,222	24,046	1,404	1,020
		ASE	1,328 (5%)	1,120 (5%)	114 (8%)	94 (9%)
Total (unique)	381	HET	24,198,160	6,654,217	12,663,914	9,549,941
		ACC	469,802	243,312	147,824	109,479
		ASE	63,541 (14%)	32,954 (14%)	19,703 (13%)	14,653 (13%)

ACC, accessible; ASE, allele-specific expression; CEU, Utah residents in the United States with Northern and Western European ancestry; CHB, Han Chinese from Beijing, China; FIN, Finnish from Finland; GBR, British in England and Scotland; HET, heterozygous; JPT, Japanese from Tokyo, Japan; MAF, minor allele frequency; SNV, single nucleotide variant; TSI, Toscani from Italy; YRI, Yorubans from Ibadan, Nigeria.

HET, ACC and ASE SNVs with MAF are shown for 381 unrelated individuals (excluding NA12878). For each of the last 3 columns, each category of HET, ACC and allele-specific SNVs is further stratified by the population MAFs: common (MAF>0.05), rare (MAF≤0.01) and very rare (MAF≤0.005). The number of allele-specific SNVs is given as a percentage of the ACC SNVs.

This table also provides the number of individuals from each ethnic population with RNA-seq data available for the ASE analyses.

**Table 2:** Breakdown of ASB SNVs in each ethnic population.

**Population**	**Individual**	**TFs**	**SNVs**	**Total**	**Common SNVs (MAF>0.05)**	**Rare SNVs (MAF≤0.01)**	**Very rare SNVs (MAF≤0.005)**
CEU	8	12	HET	10,651,149	6,308,566	2,524,103	1,783,747
			ACC	56,456	49,648	2,124	1,180
			ASB	2,802 (5%)	2,138 (4%)	264 (12%)	161 (14%)
YRI	3	4	HET	17,173,494	6,202,905	7,189,972	4,887,934
			ACC	62,525	41,365	7,129	3,508
			ASB	3,113 (5%)	1,726 (4%)	648 (9%)	422 (12%)
CHB/JPT	2	3	HET	3,185,252	2,892,601	114,990	76,669
			ACC	16,252	14,633	629	418
			ASB	598 (4%)	428 (3%)	70 (11%)	49 (12%)
Total (unique)	13	12	HET	24,198,160	6,599,592	12,659,237	9,545,352
			ACC	106,057	76,889	9,865	5,102
			ASB	6,136 (6%)	3,949 (5%)	979 (10%)	631 (12%)

ACC, accessible; ASB, allele-specific binding; CEU, Utah residents in the United States with Northern and Western European ancestry; CHB, Han Chinese from Beijing, China; HET, heterozygous; JPT, Japanese from Tokyo, Japan; MAF, minor allele frequency; SNV, single nucleotide variant; TF, transcription factor; YRI, Yorubans from Ibadan, Nigeria.

HET, ACC and ASB SNVs with MAF are shown for 381 unrelated individuals (excluding NA12878). For each of the last 3 columns, each category of HET, ACC and allele-specific SNVs is further stratified by the population MAFs: common (MAF>0.05), rare (MAF≤0.01) and very rare (MAF≤0.005). The number of allele-specific SNVs is given as a percentage of the ACC SNVs.

This table also provides the number of individuals from each ethnic population with ChIP-seq data available for the ASB analyses.

**Table 3:** Breakdown of ASB and ASE SNVs in NA12878.

**Individual**	**SNVs**	**Total**	**Common SNVs (MAF>0.05)**	**Rare SNVs (MAF≤0.01)**	**Very rare SNVs (MAF≤0.005)**
*ASE*					
NA12878	HET	1,888,613	1,739,520	44,664	24,252
	ACC	250,010	227,635	6,879	3,715
	ASE	10,011 (4%)	9,370 (4%)	219 (3%)	133 (4%)
					
*ASB*					
NA12878 (39 TFs)	HET	1,888,613	1,739,520	44,664	24,252
	ACC	48,817	44,474	1,246	643
	ASB	1,156 (2%)	1,054 (2%)	26 (2%)	10 (2%)

ACC, accessible; ASB, allele-specific binding; ASE, allele-specific expression; HET, heterozygous; MAF, minor allele frequency; SNV, single nucleotide variant.

This table shows the same HET, ACC and both ASE and ASB SNVs with MAF detected in a single individual, NA12878, who is also part of the trio family. For each of the last 3 columns, each category of HET, ACC and allele-specific SNVs is further stratified by the population MAFs: common (MAF>0.05), rare (MAF≤0.01) and very rare (MAF≤0.005). The number of allele-specific SNVs is given as a percentage of the ACC SNVs.
