# Supplementary material for: A uniform survey of allele-specific binding and expression over 1000-Genomes-Project individuals
Source: Nat Commun. 2016 Apr 18;7:11101. doi: 10.1038/ncomms11101 (PMC4837449; doi:10.1038/ncomms11101)
Supplement: Supplementary Information — Supplementary Figures 1-5, Supplementary Tables 1-5 and Supplementary Note 1 [file ncomms11101-s1.pdf]

SUPPLEMENTARY INFORMATION

Supplementary Figure 1 – 'Collapsed' enrichment analysis

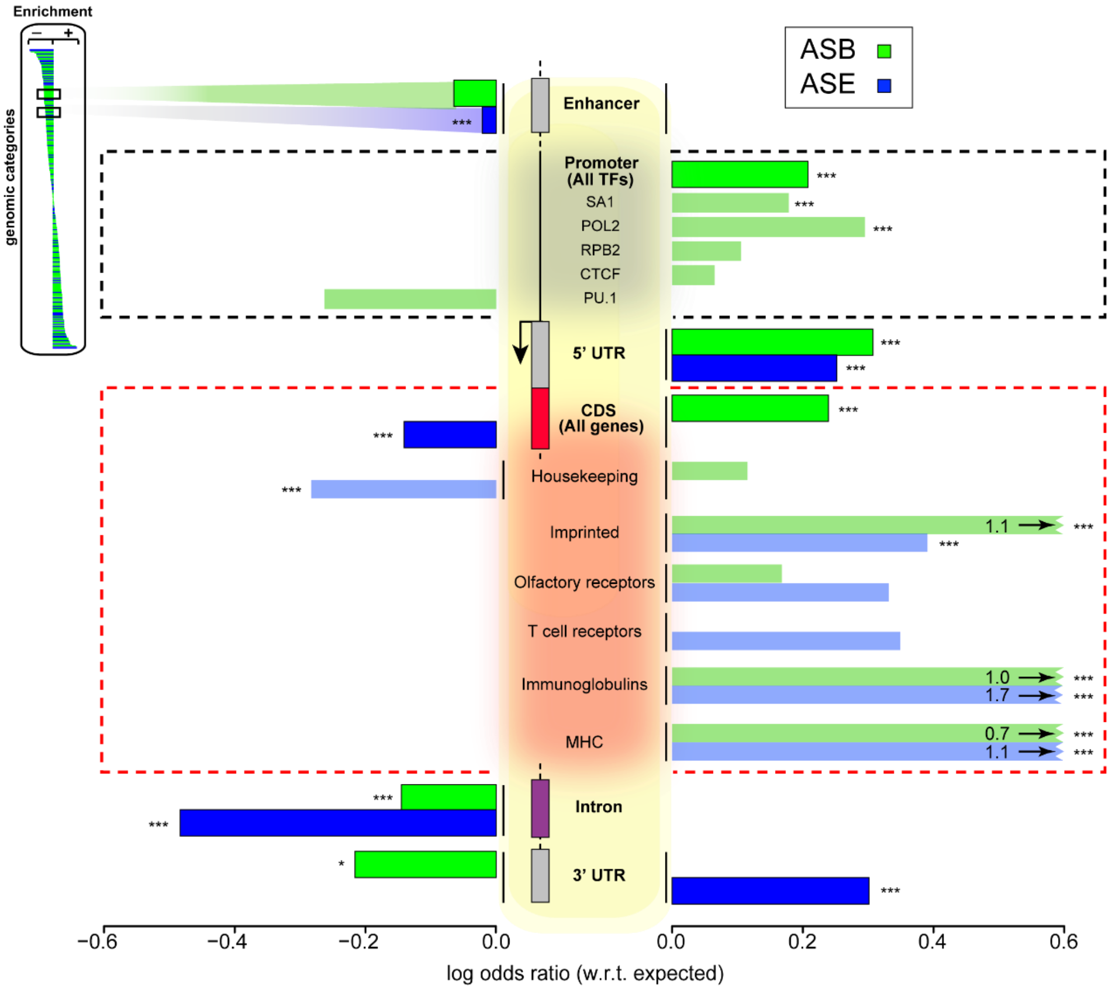

This figure shows the results for the 'collapsed' enrichment analysis. In the 'collapsed' enrichment analysis, each control or allele-specific SNV is counted once uniquely, as long as it occurs in at least one individual. We map variants associated with allele-specific binding (ASB; green) and expression (ASE; blue) to various categories of genomic annotations, such as coding DNA sequences (CDS), untranslated regions (UTRs), enhancer and promoter regions, to survey the human genome for regions more enriched in allelic behavior. Using the control non-allele-specific SNVs as the expectation, we compute the log odds ratio for ASB and ASE SNVs separately, via Fisher's exact tests. The number of asterisks depicts the degree of significance (Bonferroni-corrected): *, $p<0.05$; **, $p<0.01$; ***, $p<0.001$. For each transcription factor (TF) in AlleleDB, we also calculate the log odds ratio of ASB SNVs in promoters, providing a proxy of allele-specific regulatory role for each available TF. Genes known to be mono-allelically expressed such as imprinted and MHC genes (CDS regions) are highly enriched for both ASB and ASE SNVs. The actual log odds ratio of ASB SNVs in imprinted genes, both ASB and ASE SNVs in immunoglobulin genes and ASE SNVs for MHC genes are indicated on the bars.

Between the two enrichment analyses, we observe consistent trends in the odds ratios of ASB SNVs and ASE SNVs across the MAE gene sets, except for the T cell and olfactory receptors. The categories are enriched in ASE SNVs when we collapsed the SNV count but, interestingly, depleted when we expand the enrichment analysis in a population-aware fashion (Figure 5). This suggests that the allele-specific expression in certain T cell and olfactory receptors are not consistently observed in all individuals. Also, there is a consistent depletion in ASE SNVs for the constitutively expressed housekeeping genes, implying that most housekeeping genes give a more balanced (biallelic) expression (Figure 5).

Supplementary Figure 2 – Consistent ASB and ASE calling (by read depth)

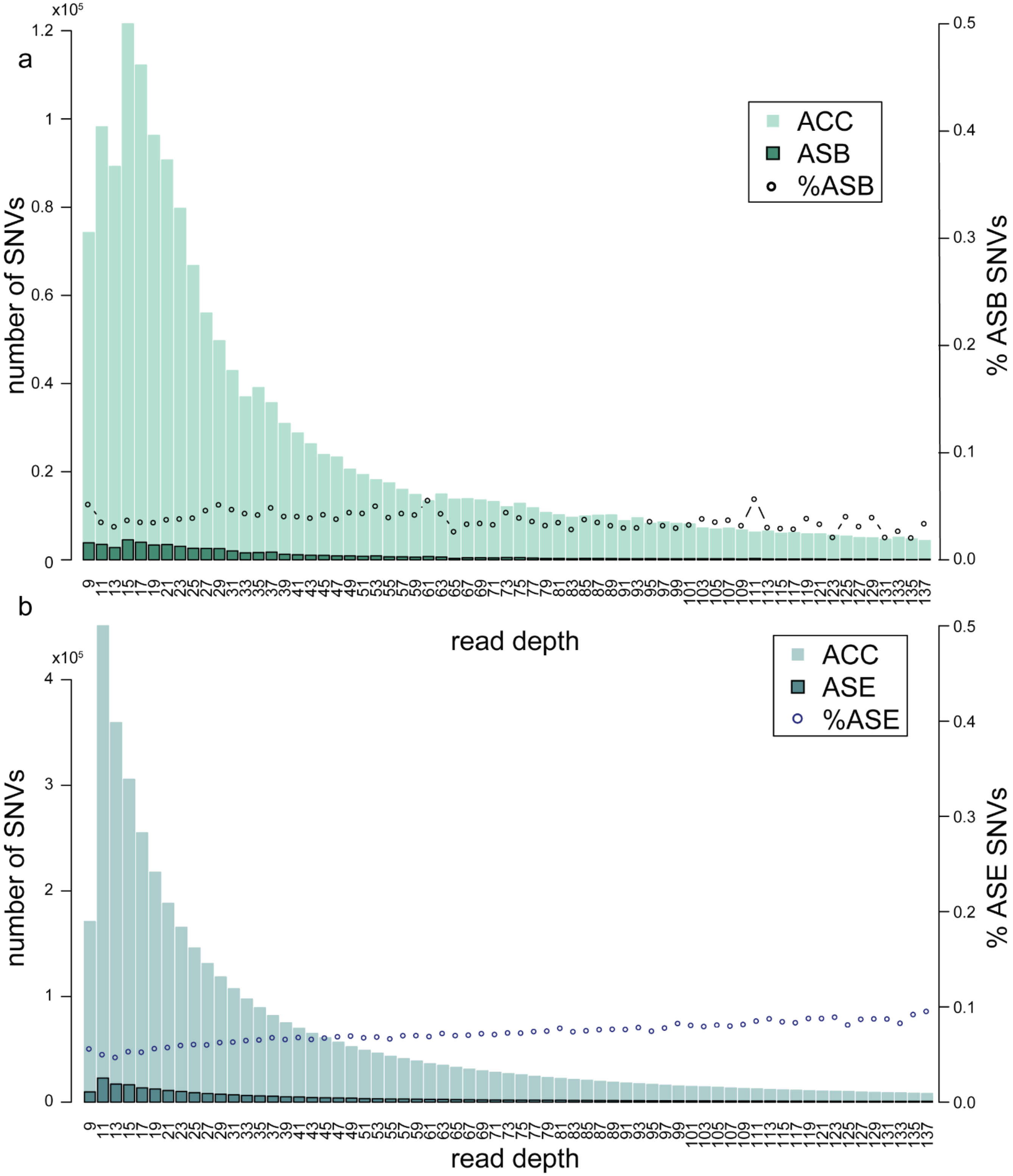

This figure shows the percentage of (a) ASB and (b) ASE SNVs (opaque bars with black boundaries) when compared to the accessible SNVs (ACC; transparent bars with no boundaries) as a function of read depth, for 381 unrelated individuals (excluding NA12878). Here, we display >90% of ASB and ASE SNVs, by not showing those with extreme read depths. Despite the bias in SNV counts towards low read depth, the percentages of our ASB and ASE SNVs that are called are relatively consistent across all read depths (% ASB or ASE; indicated by circles).

Supplementary Figure 3 – Consistent ASE calling (by ethnicity)

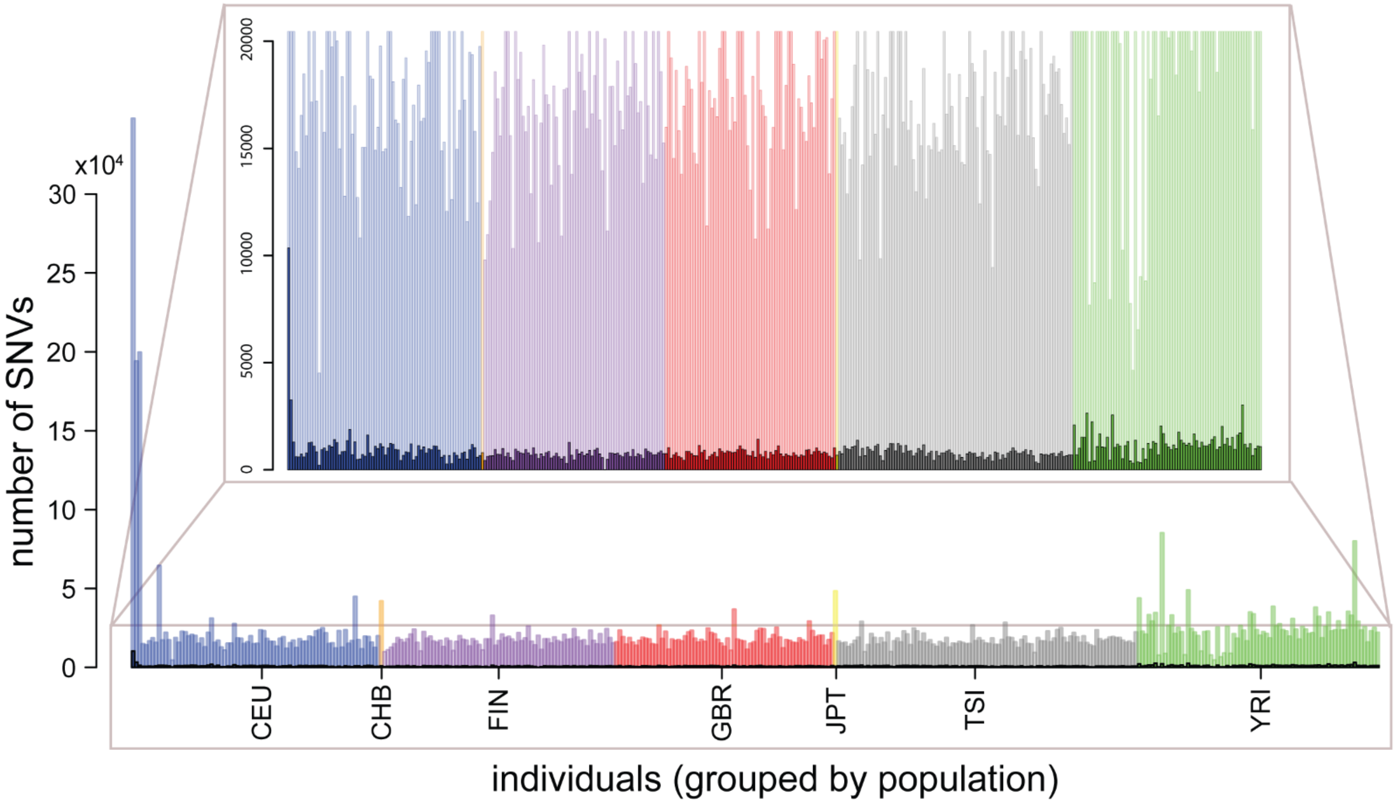

This figure shows the number of accessible (transparent-colored bars) and ASE SNVs (opaque-colored bars with black boundaries) per individual, grouped and colored by population: CEU (blue), CHB (orange), FIN (magenta), GBR (red), JPT (yellow), TSI (grey) and YRI (green). The CEU trio are represented by the three spikes at the far left. In general, the YRI have more accessible and ASE sites, probably because they have higher number of heterozygous SNVs in their genomes. The number of ASE sites in addition to the proportion with regards to their accessible sites per individual are relatively consistent.

Supplementary Figure 4 – High reproducibility of ASE calling

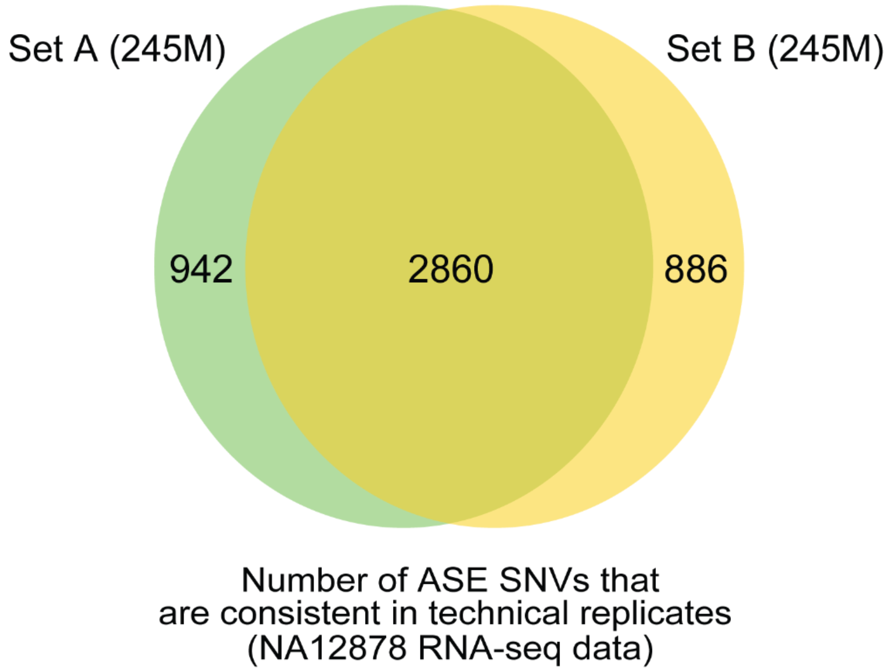

This figure shows the replication of AS calls between technical replicates. We randomly sampled two subsets of 245M (‘M’ denotes ‘million of reads’) from a pooled RNA-seq dataset of NA12878, without replacement, i.e. these two sets are mutually exclusive. We then run the AlleleDB pipeline. The Venn diagram shows that the calls between the replicates are very comparable (>75% overlap), demonstrating that our calls reproduce very well.

Supplementary Figure 5 – Replication of ASE calls with increasing read depth

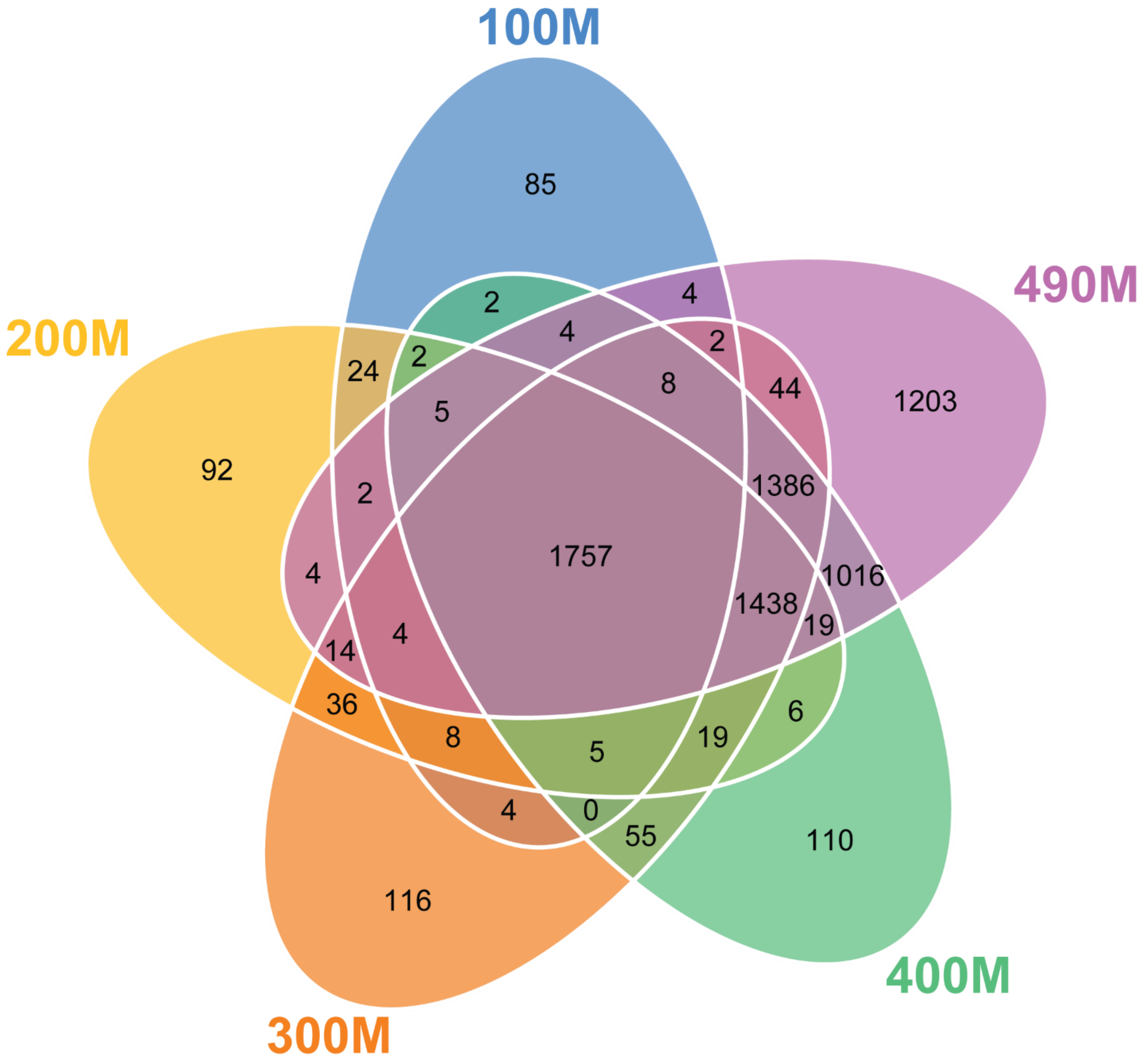

This figure shows the replication of AS calls at increasing read depths. We randomly subsampled subsets of various read coverage from a pooled RNA-seq dataset of NA12878 – 100M, 200M, 300M, 400M and 490M (‘M’ denotes ‘million of reads’) – such that each smaller pool of reads is a direct subset of the larger sets, with 490M denoting the entire set of reads. For instance, 100M is a subset of all the other sets. We then ran the AlleleDB pipeline. We show that >82% ASE sites are consistent in at least 2 subsets, with very small number of sites unique to each set.

Supplementary Table 1 – Heterogeneity of AS analyses in eight studies

Study	ASE/ASB	Reference genome	Aligner	Detection test	Filter duplicate reads (RNA-seq/ChIP-seq)	Ambiguous mapping
Montgomery et al. (2010)	ASE	Human ref genome	MAQ	Binomial test per-SNP basis	No	No*
Pickrell et al. (2010)	ASE	Human ref genome	MAQ	Betabinomial test per-gene basis	No	Yes
Lalonde et al. (2011)	ASE	--	--	--	--	--
ENCODE (2012)/ Djebali et al. (2012)	Both	Personal genome	Bowtie1	Binomial test per-SNP basis	No	No
gEUVADIS (2013)	ASE	Human ref genome	GEM mapper	Binomial test per-SNP basis	No	Yes
Kasowski et al. (2013)	Both	Personal genomes	BWA (ChIP-seq); TopHat (RNA-seq)	Binomial test per-SNP basis	Both	No
Kilpinen et al. (2013)	Both	Human ref genome	BWA	Binomial test per-SNP basis	ChIP-seq only	Yes
McVicker et al. (2013)	ASB	Human ref genome	BWA	Betabinomial test per-region basis	ChIP-seq only	Yes
AlleleDB	Both	Personal genomes	Bowtie1	Betabinomial test per-SNP basis	No	Yes

*Mapping bias in Montgomery *et al.* was deemed accounted for by weighting the binomial null with a global allelic ratio

This table shows the heterogeneity in the eight studies performing allele-specific analyses using different tools and parameters, e.g. read mapping with a range of read aligners, alignment to different reference genomes and variations of statistical tests in detecting the allele-specific variants. We uniformly processed the tools and parameters in AlleleDB.

Supplementary Table 2 – Datasets Quality Control

	**ChIP-seq datasets**	**#Filtered**	**RNA-seq datasets**	**#Filtered**	**#Total retained**
Initial	287	0	993	0	1,280
Insufficient aligned reads	276	11	987	6	1,263
Overdispersed*	186	90	955	32	1,141

*We define an "overdispersed" ChIP-seq dataset as those with $\rho \geq 0.3$, while an "overdispersed" RNA-seq dataset is defined more strictly by $\rho \geq 0.125$, which is one standard deviation more than the mean overdispersion in the RNA-seq datasets in our processing.

This table shows the number of individual datasets being flagged and segregated due to insufficient reads and due to having an "overdispersed" allelic ratio distribution.

Supplementary Table 3 – Number of reads that overlap heterozygous SNVs

Number of heterozygous SNVs	Number of **maternal** reads overlapping this number of SNVs (%)	Number of **paternal** reads overlapping this number of SNVs (%)
1	360,891 (96.866%)	360,645 (96.834%)
2	11,453 (3.074%)	11,546 (3.100%)
3	254 (0.068%)	239 (0.064%)
4	4 (0.001%)	6 (0.002%)

This table shows the number of uniquely mapped maternal (column 2) and paternal (column 3) reads that overlap a certain number of heterozygous SNVs (column 1) from an example dataset from NA12878 CTCF ChIP-seq assay. ~97% of reads that map uniquely to the maternal or paternal haplotype overlap only 1 heterozygous SNV. On average, we find that >90% of uniquely mapped reads that overlap any heterozygous SNVs at all, overlap only 1 heterozygous SNV.

Supplementary Table 4 – Heritability of allele-specific binding and expression

	**Child v Father**			**Child v Mother**			**Father v Mother**		
**ASB**	**β**	**r**	**# SNVs**	**β**	**r**	**# SNVs**	**β**	**r**	**# SNVs**
**PU.1**	1.01	0.87	33	0.98	0.97	19	0.98	0.91	13
**CTCF**	0.98	0.78	65	0.98	0.84	109	0.99	0.67	40
**ASE**	0.71	0.58	655	0.87	0.77	396	0.69	0.57	240

Child : NA12878
Father : NA12891
Mother : NA12892

This table shows the slope and Pearson's correlation results for two DNA-binding proteins, PU.1 and CTCF, and ASE for parent-child and parent-parent comparisons.

Supplementary Table 5 – Ambiguous mapping bias correction by site or read removal

**NA12878 datasets**	**Number of AS SNVs removed due to**	
	**Removal of sites with >5% allelic bias (%)**	**Removal of reads with AMB (%)**
CTCF ChIP-seq dataset (same dataset as in Supp Table 3)	20/101 (20%)	11/101 (11%)
RNA-seq dataset	17/375 (4.5%)	5/375 (1.3%)

#AMB stands for 'ambiguous mapping bias'.
*The denominators in columns 2 and 3 are the numbers of original allele-specific (AS) SNVs that are detected when AMB was not accounted for.

This table summarizes the results in examining the effects of accounting for ambiguous mapping bias via the removal of sites (column 3) and reads (column 4) using two datasets. We chose a ChIP-seq and a RNA-seq datasets from NA12878. We find that removal of sites often filters SNVs that might be still allele-specific even after removing reads that show ambiguous mapping bias (AMB), indicating that site removal can be over-conservative and read removal is able to retain AS SNVs that are still allele-specific. Also, in our study, we find that AMB seems to have a greater effect on ChIP-seq datasets. Between 10-21% of the detected AS SNVs are removed in ChIP-seq compared to 1-4% in RNA-seq datasets, depending on which bias removal strategy was adopted.

Supplementary Note 1
Alternative method to account for ambiguous mapping bias
As an alternative approach to account for ambiguous mapping bias within the personal genome framework, we also introduce some modifications into the AlleleSeq pipeline. After construction of a diploid personal genome, the reads are aligned to both haploid genomes and all valid highest scored alignments are reported for each read (allowing multi-mapping and alignments with up to two mismatches). First, similar to the original pipeline, only uniquely mapped reads are considered when the alignments are compared between the two haplotypes at all heterozygous loci. Then, for each allele with the lower count at unbalanced sites, we identify all reads (bearing the allele) that non-uniquely map to its locus on the respective haplotype. As it is not possible to unambiguously determine the origin of multimapping reads, we currently adopt the simplest approach and filter out sites with such reads. Finally, allele-specific events are then assessed for heterozygous sites that were not filtered away (additional filtering is applied to remove SNPs residing in CNV locations) by applying the beta-binomial test followed by correcting for multiple hypothesis testing.
