# Supplementary material for: A uniform survey of allele-specific binding and expression over 1000-Genomes-Project individuals
Source: Nat Commun. 2016 Apr 18;7:11101. doi: 10.1038/ncomms11101 (PMC4837449; doi:10.1038/ncomms11101)
Supplement: Supplementary Data 9 — This Word file contains the R pseudocode for the bisection method that is used to estimate the overdispersion parameter. [file ncomms11101-s10.docx]

**R pseudocode for bisection method to estimate overdispersion parameter**

while (previous_LSSE ≠ current_LSSE within 3 significant figures)

{

previous_LSSE = current_LSSE

start_ρ = prev_ρ – (prev_increment / 2)

end_ρ = prev_ρ + (prev_increment / 2)

current_increment = prev_increment / 4

range = seq(start_ρ, end_ρ, by=current_increment)

for (values in range)

{

obtain_beta-binomial_distribution

calculate_LSSE_between_beta-binomial_and_empirical_distributions

if(current_LSSE_within_for_loop > previous_LSSE_within_for_loop)

{

current_LSSE = previous_LSSE_within_for_loop

break_out_of_for_loop

}

}

}
